# Pretreatment advanced lung cancer inflammation index (ALI) for predicting early progression in nivolumab‐treated patients with advanced non–small cell lung cancer

**DOI:** 10.1002/cam4.1234

**Published:** 2017-11-18

**Authors:** Takayuki Shiroyama, Hidekazu Suzuki, Motohiro Tamiya, Akihiro Tamiya, Ayako Tanaka, Norio Okamoto, Kenji Nakahama, Yoshihiko Taniguchi, Shun‐ichi Isa, Takako Inoue, Fumio Imamura, Shinji Atagi, Tomonori Hirashima

**Affiliations:** ^1^ Department of Thoracic Oncology Osaka Habikino Medical Center Osaka Japan; ^2^ Department of Thoracic Oncology Osaka International Cancer Institute Osaka Japan; ^3^ Department of Internal Medicine Kinki‐chuo Chest Medical Center Osaka Japan; ^4^ Department of Clinical Research Center Kinki‐chuo Chest Medical Center Osaka Japan

**Keywords:** Advanced lung cancer inflammation index, early progression, neutrophil‐to‐lymphocyte ratio, nivolumab, progression‐free survival

## Abstract

Programmed death‐ligand 1 (PD‐L1) expression status is inadequate for indicating nivolumab in patients with non–small cell lung cancer (NSCLC). Because the baseline advanced lung cancer inflammation index (ALI) is reportedly associated with patient outcomes, we investigated whether the pretreatment ALI is prognostic in NSCLC patients treated with nivolumab. We retrospectively reviewed the medical records of all patients treated with nivolumab for advanced NSCLC between December 2015 and May 2016 at three Japanese institutes. Multivariate logistic regression and Cox proportional hazards models were used to assess the impact of the pretreatment ALI (and other inflammation‐related parameters) on progression‐free survival (PFS) and early progression (i.e., within 8 weeks after starting nivolumab). A total of 201 patients were analyzed; their median age was 68 years (range, 27–87 years), 67% were men, and 24% had an Eastern Cooperative Oncology Group (ECOG) performance status of 2 or higher. An ECOG performance status ≥2, serum albumin <3.7 g/dL, neutrophil‐to‐lymphocyte ratio ≥4, and ALI <18 were significantly associated with poor PFS and early progression on univariate analysis. Multivariate analyses revealed that pretreatment ALI <18 was independently associated with inferior PFS (median, 1.4 vs. 3.7 months, *P* < 0.001) and a higher likelihood of early progression (odds ratio, 2.76; 95% confidence interval 1.44–5.34; *P* = 0.002). The pretreatment ALI was found to be a significant independent predictor of early progression in patients with advanced NSCLC receiving nivolumab, and may help identify patients likely to benefit from continued nivolumab treatment in routine clinical practice.

## Introduction

Immunotherapy represents a major breakthrough in cancer treatment. Nivolumab is a fully humanized immunoglobulin G4 antibody that inhibits programmed death‐1 (PD‐1), a T‐cell checkpoint receptor protein. Nivolumab disrupts the interaction of PD‐1 with its ligands PD‐L1 and PD‐L2. In two recent phase III studies 1, 2, nivolumab showed a survival benefit over docetaxel in patients who were previously treated for advanced non–small cell lung cancer (NSCLC). While PD‐L1 expression does correlate with treatment outcomes, whether its expression status is the optimal biomarker for indicating nivolumab in lung cancer patients remains unclear 3, 4. Additional novel biomarkers predictive of nivolumab efficacy that can complement PD‐L1 expression status are required to identify patients who will derive durable responses to nivolumab treatment.

Tumor progression is suggested to be closely associated with cancer‐related inflammatory and nutritional statuses 5, 6, 7. Previous studies have identified several inflammation/nutrition biomarkers as prognostic factors; these include body mass index (BMI) 8, 9, C‐reactive protein (CRP) 10, 11, 12, CRP‐to‐albumin (ALB) ratio (CAR) 13, 14, and neutrophil‐to‐lymphocyte ratio (NLR) 15, 16, 17, 18. Additionally, the advanced lung cancer inflammation index (ALI) at baseline has recently been shown to be an independent predictor of survival in patients with advanced NSCLC 19, 20, 21. Nevertheless, the roles of these measures in predicting survival and early progression in patients with NSCLC who were treated with nivolumab have not been determined.

Therefore, we analyzed the impact of ALI and other markers of inflammation on nivolumab‐treated patients with NSCLC in routine clinical practice.

## Patients and Methods

### Patient eligibility

We conducted a retrospective multicenter study of all patients with previously treated advanced NSCLC who initiated nivolumab treatment (3 mg/kg intravenously every 2 weeks) at three institutes in Japan between December 17, 2015 (the date nivolumab was approved in Japan) and May 31, 2016. Patients were excluded from our analysis if they received nivolumab as part of a clinical trial or concurrently received any treatment with other anticancer therapies. This study was approved by the appropriate Ethical Review Board committee of each participating institution, and was registered at the UMIN Clinical Trials Registry (UMIN000025908). The research was conducted in accordance with the 1964 Declaration of Helsinki and its later amendments. All participants provided informed written consent prior to enrollment in this study.

### Definition of early progression

Progressive disease was defined according to Response Evaluation Criteria in Solid Tumors version 1.1; cancer‐related death was considered progression. We defined early progression as that occurring within 8 weeks after starting nivolumab treatment; this was based on the median response times to nivolumab treatment in two previous phase III studies, which was approximately 2 months for each 1, 2.

### Data collection

We retrospectively reviewed medical records and collected data that included patient demographics, Eastern Cooperative Oncology Group Performance Status (ECOG PS), smoking history, histology, molecular profiling for epidermal growth factor receptor and anaplastic lymphoma kinase when available, previous treatments, history of pulmonary disease, nutritional status including body weight and BMI, and baseline laboratory findings (within 2 weeks prior to the initiation of nivolumab) including complete blood count, CRP, serum ALB, CAR, NLR, and ALI. The ALI score was calculated as BMI×ALB/NLR, and patients with a low ALI score were suspected of having high systemic inflammation. The response of nivolumab was determined by the Response Evaluation Criteria in Solid Tumors version 1.1, and dates of progression as well as of death or last follow‐up were specified. All clinical data were confirmed by an independent contract research organization (EP‐SOGO Co., Ltd.). The cut‐off date for data collection was September 30, 2016.

### Statistical analyses

Based on previous reports, the cut offs for CRP, NLR, and ALI were defined as 1.0 mg/dL 10, 4 15, and 18 19, respectively. The cut offs for other continuous variables were defined using their median values. Survival curves were analyzed using the Kaplan–Meier method, and the differences between the groups were compared by using the log‐rank test. Univariate and multivariate analyses were performed using the Cox proportional hazards and logistic regression models. Continuous variables were analyzed using the Mann–Whitney *U* test, while categorical variables were analyzed using Fisher's exact test. All *P*‐values were based on the two‐sided hypothesis, and a *P* < 0.05 was considered statistically significant. All statistical analyses were conducted using the R software (version 3.2.0). All variables with a *P* < 0.05 on univariate analysis were included in the multivariate model; however, considering the linearly dependent relationships among these variables (as ALI is calculated using ALB and NLR), the ALB and NLR were excluded from multivariate analysis if ALI remained a significantly stronger predictor than the two other factors on univariate analysis.

## Results

### Patients

A total of 201 patients who were previously treated for advanced NSCLC received nivolumab between December 17, 2015 and May 31, 2016. The baseline clinicopathological characteristics of the patients are summarized in Table 1. The median age at the time of nivolumab treatment was 68 years (range: 27–87 years); 67% of the patients were men, 24% had an ECOG PS of 2 or higher, and the vast majority had nonsquamous histology and a history of smoking. The median baseline NLR was 3.3, with an NLR <4 in 60.7% of patients and an NLR ≥4 in 39.3%. The median baseline ALI was 22.7, with an ALI <18 in 36.3% of patients and an ALI ≥18 in 63.7%. The median follow‐up period was 12.4 months (Kaplan–Meier estimate).

**Table 1:** Patient characteristics at baseline (*n* = 201)

	*n* (%)
Age	
Median (range)	68 (27–87)
Gender	
Male	135 (67.2%)
Female	66 (32.8%)
ECOG performance status	
0–1	153 (76.1%)
2–4	48 (23.9%)
Histology	
Squamous cell carcinoma	41 (20.4%)
Nonsquamous cell carcinoma	160 (79.6%)
Smoking status	
Never smoker	44 (21.9%)
Current or former smoker	157 (78.1%)
No. of prior therapies	
Median (range)	2 (1–10)
Body mass index, kg/m^2^	
Median (range)	21.2 (9.4–37.8)
<18.5	48 (23.9%)
18.5–24.99	120 (59.7%)
≥25	33 (16.4%)
C‐reactive protein, mg/dL	
Median (range)	0.7 (0.01–30.3)
Serum albumin^a^, g/dL	
Median (range)	3.7 (2.1–4.9)
C‐reactive protein‐to‐albumin ratio^a^	
Median (range)	0.17 (0.002–13.8)
Neutrophil‐to‐lymphocyte ratio	
Median (range)	3.3 (0.70–71.4)
<4	122 (60.7%)
≥4	79 (39.3%)
Advance lung cancer inflammation index^a^	
Median (range)	22.7 (0.7–133.5)
<18	69 (36.3%)
≥18	121 (63.7%)

ECOG, Eastern Cooperative Oncology Group.

a Data were unavailable in 11 cases.

### Survival

**Figure 1:**
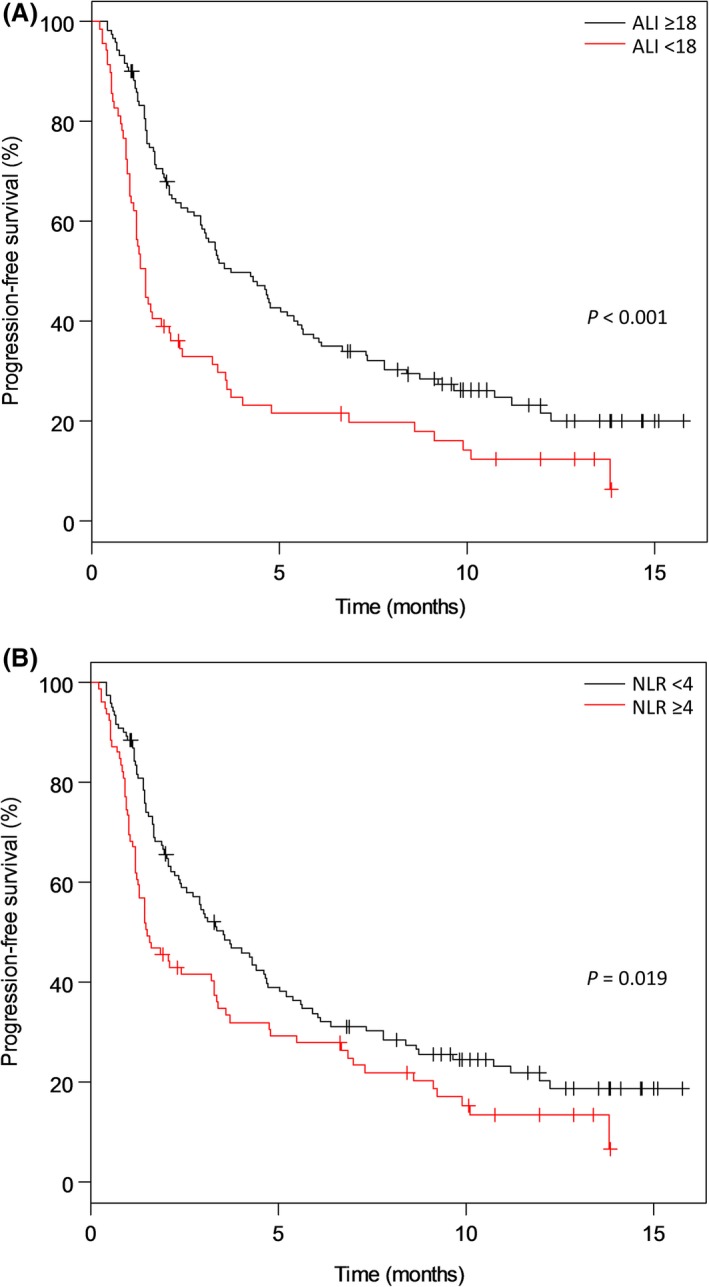
Kaplan–Meier curves of progression‐free survival. A pretreatment advanced lung cancer inflammation index (ALI) <18 (A) and neutrophil‐to‐lymphocyte ratio (NLR) ≥4 (B) were independently associated with inferior progression‐free survival.

At the time of data cut off, 158 patients (78.6%) had progressed and 92 (45.8%) had died. The median PFS was 2.9 months (95% confidence interval [CI]: 2.1–3.7 months). According to Kaplan–Meier analysis, patients with ALIs <18 experienced significantly worse PFS than those with ALIs ≥18 (1.4 vs. 3.7 months, respectively, *P* < 0.001) (Fig. 1A). Additionally, patients with NLRs ≥4 had significantly worse PFS rates than those with NLRs <4 (1.5 vs. 3.5 months, respectively, *P* = 0.019) (Fig. 1B). On univariate analysis, factors significantly associated with poor PFS included PS ≥2, CAR >0.17, NLR ≥4, and ALI <18 (Table 2). Multivariate analysis of PS, CAR, and ALI revealed that a PS ≥2 and an ALI <18 were associated with poor PFS (Table 2).

**Table 2:** Prognostic factors associated with progression‐free survival as determined by univariate and multivariable analyses

	Univariate analysis		Multivariate analysis	
	HR (95% CI)	*P*‐value	HR (95% CI)	*P*‐value
Age <75 years	1.28 (0.87–1.89)	0.21		
Female gender	1.34 (0.97–1.85)	0.077		
ECOG PS ≥2	1.83 (1.29–2.61)	<0.001	1.60 (1.10–2.33)	0.013
Squamous histology	1.25 (0.85–1.83)	0.26		
Never smoker	1.36 (0.95–1.94)	0.096		
No. of prior therapies ≥2	1.18 (0.86–1.63)	0.31		
Body mass index <18.5 kg/m^2^	1.14 (0.80–1.64)	0.46		
C‐reactive protein >1.0 mg/dL	1.37 (1.00–1.88)	0.051		
Serum albumin <3.7 g/dL	1.75 (1.26–2.44)	<0.001		
CAR >0.17	1.39 (1.01–1.92)	0.044	1.24 (0.89–1.73)	0.20
NLR ≥4	1.46 (1.06–2.00)	0.020		
ALI <18	1.83 (1.31–2.54)	<0.001	1.72 (1.24–2.41)	0.001

HR, hazard ratio; CI, confidence interval; ECOG PS, Eastern Cooperative Oncology Group performance status; CAR, C‐reactive protein‐to‐albumin ratio; NLR, neutrophil‐to‐lymphocyte ratio; ALI, advanced lung cancer inflammation index.

### Response

The overall response rate was 15.9% (95% CI: 11.2–21.7%); a total of 85 patients (42.3%) experienced early progression. Univariate analysis revealed that a PS ≥2, CRP >1.0 mg/dL, ALB <3.7 g/dL, NLR ≥4, and ALI <18 were associated with a higher likelihood of early progression (Table 3). The final logistic regression model included PS, CRP, and ALI; among these factors, only ALI was significantly associated with early progression on multivariate analysis (adjusted odds ratio 2.76, 95% CI: 1.44–5.34, *P* = 0.002) (Table 3).

**Table 3:** Prognostic factors associated with early progression as determined by univariate and multivariable analyses

	Univariate analysis		Multivariate analysis	
	Odds ratio (95% CI)	*P*‐value	Odds ratio (95% CI)	*P*‐value
Age <75 years	1.45 (0.79–2.63)	0.21		
Female gender	1.22 (0.67–2.17)	0.53		
ECOG PS ≥2	2.64 (1.36–5.14)	0.004	2.02 (0.99–4.19)	0.055
Squamous histology	1.39 (0.70–2.77)	0.35		
Never smoker	1.18 (0.60–2.33)	0.63		
No. of prior therapies ≥2	1.46 (0.82–2.63)	0.20		
Body mass index <18.5 kg/m^2^	1.03 (0.54–2.00)	0.92		
C‐reactive protein >1.0 mg/dL	1.87 (1.06–3.31)	0.031	1.28 (0.67–2.44)	0.45
Serum albumin <3.7 g/dL	2.50 (1.37–4.55)	0.003		
CAR >0.17	1.61 (0.89–2.94)	0.11		
NLR ≥4	2.28 (1.27–4.06)	0.005		
ALI <18	3.27 (1.78–6.11)	<0.001	2.76 (1.45–5.34)	0.002

Early progression was defined as progression occurring within 8 weeks after starting treatment of nivolumab. CI, confidence interval; ECOG PS, Eastern Cooperative Oncology Group performance status; CAR, C‐reactive protein‐to‐albumin ratio; NLR, neutrophil‐to‐lymphocyte ratio; ALI, advanced lung cancer inflammation index.

**Figure 2:**
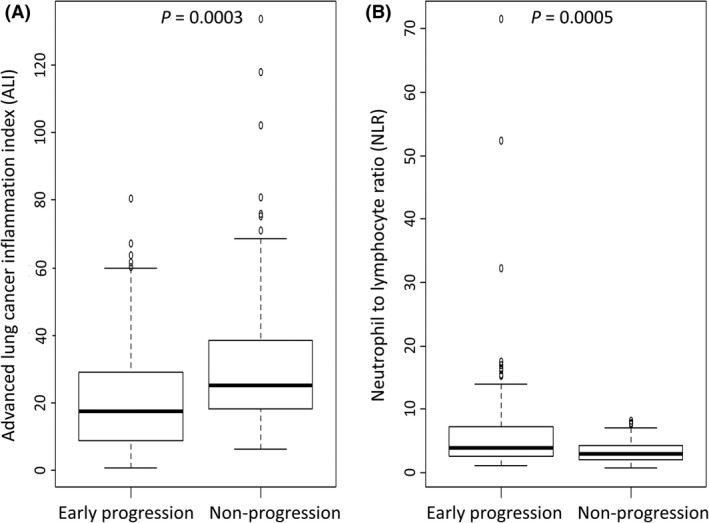
Box plots representing the pretreatment advanced lung cancer inflammation index (ALI) (A) and neutrophil‐to‐lymphocyte ratio (NLR) (b) in patients with early progression versus those without early progression. Early progression was defined as that occurring within 8 weeks after commencing treatment with nivolumab. The horizontal bars represent the median values.

The distributions of pretreatment ALIs and NLRs for patients with and without early progression are shown in Figure 2. Pretreatment ALI was significantly lower in patients who experienced early progression than in those who did not (*P* = 0.0003) (Fig. 2A). The pretreatment NLR was significantly higher in patients who experienced early progression than in those who did not (*P* = 0.0005) (Fig. 2B).

### Association of ALI with clinicopathological features

A total of 69 patients (36.3%) had a pretreatment ALI of <18, while 121 patients (63.7%) had a pretreatment ALI of ≥18. We compared the clinicopathological characteristics between the two groups (Table 4); the continuous variables BMI, CRP, CAR, serum ALB, and NLR all had significantly worse scores in patients of the ALI <18 group (*P* < 0.001).

**Table 4:** Patient characteristics at baseline classified by the advanced lung cancer inflammation index (*n* = 190)

	ALI <18 (*n* = 69)	ALI ≥18 (*n* = 121)	*P*‐value
Age			
Median (range)	67 (27–87)	69 (45–85)	0.30
Gender			0.42
Male	43 (62.3%)	83 (68.6%)	
Female	26 (37.7%)	38 (31.4%)	
ECOG PS			0.008
0–1	45 (65.2%)	100 (82.6%)	
2–4	24 (34.8%)	21 (17.4%)	
Histology			0.85
Squamous cell carcinoma	14 (20.3%)	23 (19.0%)	
Nonsquamous cell carcinoma	55 (79.7%)	98 (81.0%)	
Smoking status			0.47
Never smoker	18 (26.1%)	25 (20.7%)	
Current or former smoker	51 (73.9%)	96 (79.3%)	
Body mass index, kg/m^2^			
Median (range)	18.8 (9.4–27.1)	22.2 (12.8–37.8)	<0.001
C‐reactive protein, mg/dL			
Median (range)	1.67 (0.01–30.3)	0.44 (0.01–9.90)	<0.001
Serum albumin^a^, g/dL			
Median (range)	3.4 (2.1–4.8)	3.8 (2.6–4.9)	
C‐reactive protein‐to‐albumin ratio^a^			<0.001
Median (range)	0.51 (0.003–13.8)	0.12 (0.002–3.08)	
Neutrophil‐to‐lymphocyte ratio			<0.001
Median (range)	6.19 (2.52–71.4)	2.53 (0.70–5.25)	<0.001
Advance lung cancer inflammation index^a^			
Median (range)	10.16 (0.74–17.65)	30.02 (18.18–133.5)	<0.001

ALI, advanced lung cancer inflammation index; ECOG PS, Eastern Cooperative Oncology Group performance status.

a Data were unavailable in 11 cases.

## Discussion

We found that the pretreatment ALI was found to be a strong independent predictor of PFS and early progression in patients with previously treated NSCLC who received nivolumab. ECOG PS, serum ALB, NLR, and ALI were also associated with PFS and early progression. Our results suggest that these inflammatory and nutritional parameters may serve as predictive markers for survival and response in nivolumab‐treated patients with advanced NSCLC.

Immune checkpoint inhibitors enhance antitumor immunity by blocking the negative regulation of T‐cell activation, which promotes the immune system's ability to attack cancer cells. Systemic inflammation plays a key role in tumor survival, proliferation, angiogenesis, immunosuppression, and nutritional depletion 6, 22. Nutritional status is an important factor in immune responses, and malnutrition is a main cause of immunodeficiency 23. Cancer‐associated inflammation and malnutrition are key determinants not only of tumor progression but also of the response to immunotherapy 6, 7, 24. Therefore, the efficacy of nivolumab treatment is thought to be heavily dependent on the patients' inflammatory status at baseline.

Several studies have shown that pretreatment NLR correlates with the outcomes of ipilimumab‐treated melanoma patients 16, 17, 18, 25. Recently, a retrospective analysis of 175 patients with NSCLC treated with nivolumab revealed that pretreatment NLR was independently associated with inferior outcomes 26. Our findings are generally consistent with those of earlier studies. On the other hand, no information is currently available regarding the relationship between the ALI and outcomes of patients treated with immunotherapy. To our knowledge, this is the first study to investigate the role of ALI as a risk factor for early progression in NSCLC patients treated with nivolumab in routine practice. The ALI was devised to assess the degree of systemic inflammation in patients with advanced NSCLC 19, and a low ALI is indicative of high systemic inflammation. Combined with the BMI, serum ALB, and NLR, we found that the ALI serves as a more comprehensive indicator of outcome, and thus plays an important role as a predictor of efficacy in patients treated with nivolumab.

Tumor progression that occurs relatively soon after the initiation of nivolumab can cause indecision among clinicians regarding whether to continue administering nivolumab. If there is no deterioration in ECOG PS, clinicians must consider whether tumor progression is in fact occurring or whether the detected feature is actually pseudoprogression, despite the latter's low likelihood. Such a dilemma can be exasperated when patients request the continuation of nivolumab treatment. The importance of predicting early progression is to help clinicians screen for patients who are unlikely to benefit from nivolumab and to judge whether ceasing nivolumab and switching to another treatment is warranted. On multivariate analysis, the ALI was found to be a more powerful predictor of early progression than ECOG PS. Therefore, the ALI can be used as a simple index for monitoring the therapeutic effects of nivolumab, and for detecting early progression within 8 weeks after initiation of nivolumab in patients with advanced NSCLC during follow‐up. Our data suggest that the discontinuation of nivolumab treatment in patients with high ALI scores may be justified.

Biomarkers such as PD‐L1, tumor‐infiltrating immune cells, and tumor mutation burden have emerged as potential predictors of the response to nivolumab. A recent study evaluating objective determination of PD‐L1 expression in NSCLC revealed heterogeneity within these tumors 27. Regarding tumor‐infiltrating immune cells, a recent study that investigated the proportions of malignant and immune cells expressing PD‐L1 in patients with NSCLC showed poor concordance in the scoring of immune cells among pathologists 28. In an exploratory analysis of CheckMate 026 that compared first‐line nivolumab with chemotherapy in patients with PD‐L1‐positive NSCLC, patients with a high tumor mutation burden had a higher response rate with nivolumab than with chemotherapy 29. Tumor mutation burden can help predict responses to immunotherapies, however, whole‐exome sequencing is an expensive, time‐consuming technique that is presently unavailable in wide clinical practice. Given these circumstances, ALI assessment is an alternative that can be performed rapidly and precisely by a simple and inexpensive blood test; our data support the use of ALI assessment in routine clinical practice.

There are several limitations in our study. First, our conclusions are subject to the caveats inherent to our study being retrospective. Second, this study focused on the pretreatment ALI and NLR, which may be affected by other factors like infections or cancer‐related complications. Finally, because of the lack of information regarding PD‐L1 expression in patients of our cohort, we were unable to include PD‐L1 status as a variable in our analyses.

In conclusion, we revealed the pretreatment ALI to be a strong independent predictor of early progression in patients with advanced NSCLC who received nivolumab. As ALI assessment appears to be easily calculated following widely available tests without additional costs, this parameter can help identify patients with poor prognoses and assist in clinical decision making regarding the continuation of nivolumab treatment. Further prospective studies are warranted to validate our findings.

## Conflict of Interest

None declared.

## Supporting information


**Table S1.** Progression‐free survival in all patients and in patients who experienced early progression.
