# Supplementary material for: Pretreatment advanced lung cancer inflammation index (ALI) for predicting early progression in nivolumab‐treated patients with advanced non–small cell lung cancer
Source: Cancer Med. 2017 Nov 18;7(1):13–20. doi: 10.1002/cam4.1234 (PMC5773945; doi:10.1002/cam4.1234)
Supplement: Supplementary file 1 — Table S1. Progression‐free survival in all patients and in patients who experienced early progression. [file CAM4-7-13-s001.docx]

**Supporting Information**

Additional supporting information may be found in the online version of this article:

**Table S1.** Progression-free survival in all patients and in patients who experienced early progression.

|  | Overall | Early progression |
| --- | --- | --- |
|  | months (95%CI) | days (95%CI) |
| Age  <75  ≥75 | 2.40 (1.90–3.53)  3.73 (2.07–8.67) | 32.0 (28.3–33.9)  38.5 (25.2–41.5) |
| Sex  Female  Male | 2.07 (1.50–3.33)  3.33 (2.10–4.60) | 33.5 (27.4–36.6)  32.0 (27.8–34.7) |
| ECOG PS  ≥2  0,1 | 1.53 (1.17–3.20)  3.30 (2.40–4.70) | 33.0 (28.8–34.2)  33.0 (29.0–35.3) |
| Histology  Squamous  Non-squamous | 1.90 (1.47–3.33)  3.27 (2.23–4.40) | 28.5 (24.1–35.8)  34.0 (28.9–35.1) |
| Smoking status  Never smoker  Current or former smoker | 2.10 (1.43–4.70)  3.20 (2.07–4.30) | 29.5 (24.6–36.3)  33.0 (28.7–34.9) |
| No. of prior therapies  ≥2  <2 | 2.40 (1.67–3.70)  3.20 (2.07–4.63) | 34.0 (29.4–36.2)  28.0 (24.5–33.6) |
| Body mass index, kg/m^2^  <18.5  ≥18.5 | 2.40 (1.50–4.70)  2.93 (2.07–4.23) | 32.5 (22.5–36.2)  33.0 (29.3–35.1) |
| C-reactive protein, mg/dL  >1.0  ≤1.0 | 2.03 (1.30–3.33)  3.40 (2.40–4.80) | 28.0 (23.2–30.8)  38.0 (32.7–39.5) |
| Serum albumin, g/dL  <3.7  ≥3.7 | 1.83 (1.43–2.90)  4.70 (3.20–6.83) | 28.0 (24.3–31.8)  36.0 (32.9–40.1) |
| CAR  >0.17  ≤0.17 | 2.07 (1.43–3.33)  3.57 (2.90–5.60) | 28.0 (24.4–31.8)  37.0 (32.4–40.0) |
| NLR  ≥4  <4 | 1.50 (1.27–3.33)  3.53 (2.53–4.67) | 30.0 (25.6–33.1)  34.0 (29.8–37.6) |
| ALI  <18  ≥18 | 1.43 (1.17–2.10)  3.70 (3.00–5.47) | 29.0 (24.5–32.3)  35.0 (31.0–38.6) |

Early progression was defined as progression occurring within 8 weeks after starting treatment of nivolumab. Abbreviations: CI, confidence interval; ECOG PS, Eastern Cooperative Oncology Group performance status; CAR, C-reactive protein-to-albumin ratio; NLR, neutrophil-to-lymphocyte ratio; ALI, advanced lung cancer inflammation index.
